# Concurrent evaluation of independently cued features during perceptual decisions and saccadic targeting in visual search

**DOI:** 10.3758/s13414-019-01854-w

**Published:** 2019-09-09

**Authors:** Doug J. K. Barrett, Oliver Zobay

**Affiliations:** 1 Department of Neuroscience, Psychology and Behaviour, University of Leicester, Leicester, LE1 9HN UK; 2 Hearing Sciences, University of Nottingham, Nottingham, UK

**Keywords:** Visual search, Selection, Decision, Capacity

## Abstract

Simultaneous search for one of two targets is slower and less accurate than search for a single target. Within the Signal Detection Theoretic (SDT) framework, this can be attributed to the division of resources during the comparison of visual input against independently cued targets. The current study used one or two cues to elicit single- and dual-target searches for orientation targets among similar and dissimilar distractors. In Experiment 1, the accuracy of target discrimination in brief displays was compared at setsizes of 1, 2 and 4. Results revealed a reduction in accuracy that scaled with the product of set size and the number of cued targets. In Experiment 2, the accuracy and latency of observers’ saccadic targeting were compared. Fixations on single-target searches were highly selective towards the target. On dual-target searches, the requirement to detect one of two targets produced a significant reduction in target fixations and equivalent rates of fixations to distractors with opposite orientations. For most observers, the dual-target cost was predicted by an SDT model that simulated increases in decision-noise and the distribution of capacity-limited resources during the comparison of selected input against independently cued targets. For others, search accuracy was consistent with a single-item limit on perceptual decisions and saccadic targeting during search. These findings support a flexible account of the dual-target cost based on different strategies to resolve competition between independently cued targets.

**Electronic supplementary material:**

The online version of this article (10.3758/s13414-019-01854-w) contains supplementary material, which is available to authorized users.

## Introduction

Finding a target-object in the scene requires observers to compare visual input at different locations with an internal representation of the target’s features. This ability is thought to rely on components of selective attention that integrate information across frontal, parietal and visual cortical regions (Ptak, 2012). Prior to search, an attentional template in visual short-term memory maintains a description of the target’s visual features (i.e., it’s colour and orientation or categorical identity: Carlisle, Arita, Pardo, & Woodman, 2011; Duncan & Humphreys, 1989; Desimone & Duncan, 1995; Olivers, Peters, Houtkamp, & Roelfsema, 2011). During search, information in the attentional template is used to weight relevant over irrelevant visual input across the visual field (Chelazzi, Duncan, Miller, & Desimone, 1998; Martinez-Trujillo & Treue, 2004; Maunsell & Treue, 2006). This top-down control-signal mediates interactions in topographical cortices that represent the locations of target features (Corbetta, Miezin, Dobmeyer, Shulman, & Petersen, 1990; Ester, Sutterer, Serences, & Awh, 2016; Ling, Liu, & Carrasco, 2009; Müller et al., 2006). Within a single fixation, selective attention protects capacity-limited decision processes by restricting the evaluation of visual input to locations containing task-relevant features (Bundesen, Habekost, & Kyllingsbæk, 2005; Eimer, 2015; Folk, Remington, & Wright, 1994; Wolfe & Gray, 2007). When search operates over longer durations, selection also informs the programming of saccades to locations that are most likely to contain the target (Bisley & Goldberg, 2010; Rutishauser & Koch, 2007; Zelinsky & Bisley, 2015). Within this framework, the accuracy of perceptual decisions and saccadic sampling depend on the specificity of the information that informs selection, as well as the decision processes that operate on objects at selected locations (Eimer, 2015; Palmer, 1995).

Most studies of search have focussed on the accuracy and speed of detection for a single target. In this situation, selection elicits a topographic map that represents objects in terms of their similarity to the target’s features (Zelinsky & Bisley, 2015). In many situations, however, observers are required to detect more than one possible target. Airport baggage screeners, for example, search for different types of objects (e.g. guns, knives or aerosols). Previous research has shown that this type of search is difficult: Search for one of two targets is often slower and less accurate than separate searches for the same two targets. This ‘dual-target’ cost has been reported for targets that differ from non-targets (distractors) on a value within a single feature dimension (i.e., colour), as well as a conjunction of values from different feature dimensions (i.e., colour and orientation; Kaplan & Carvellas, 1965; Menneer, Barrett, Phillips, Donnelly, & Cave, 2007; Menneer, Cave, & Donnelly, 2009; Wing & Allport, 1972). The dual-target cost has been reported in brief displays that preclude eye movements (Houtkamp & Roelfsema, 2009) and free-view displays, where the accuracy of saccadic sampling appears to be reduced compared to single-target searches (Dombrowe, Donk, & Olivers, 2011; Stroud, Menneer, Cave, & Donnelly, 2012). Whether these findings reflect a limit on the number of attentional templates that can inform selection at any one time, or post-selection limits on the evaluation of selected objects against multiple decision criteria, is currently unknown.

In the studies above, the dual-target cost describes a reduction in performance associated with the requirement to detect one of two possible targets. In this context, ‘single-‘ and ‘dual-target search’ denote the number of cued values the observer must evaluate to detect the presence or absence of a single target. As such, the terms reflect changes to the observer’s attentional set rather than the number of targets that can appear in the display (Barrett & Zobay, 2014; Cave, Menneer, Nomani, Stroud, & Donnelly, 2018; Irons,Folk & Remington, 2012; Stroud et al., 2012). One explanation for the dual-target cost is that selection during search is limited to a single-item attentional template (SIT: Beck & Hollingworth, 2017). Evidence consistent with this account was reported by Houtkamp and Roelfsema (2009), who used a rapid serial presentation (RSVP) task to compare detection accuracy for briefly presented targets preceded by one or two cues. Their data revealed a reliable decrease in detection accuracy for targets preceded by two cues, which was best accounted for by a signal-detection (SDT) model that limited search to a single target. Support for a SIT limit on selection has also been obtained by studies investigating the effects of visual short-term memory (VSTM) load on search. For example, van Moorselaar and colleagues (van Moorselaar, Theeuwes, & Olivers, 2014) required observers to remember a variable number of colours prior to onset of search display. At a VSTM load of one, their data revealed a significant increase in response times (RTs) when the colour of the memory item matched that of a distractor during search. When memory displays contained more than one remembered colour, however, search RTs were unaffected (see Hollingworth & Beck, 2016, for contradictory evidence). Based on these and similar results, Olivers and colleagues (Olivers et al., 2011) proposed a functional distinction between objects maintained in VSTM. During search, the attentional template has direct access to the mechanisms that select relevant visual input. Accessory objects, which are outside the current focus of attention, are maintained in a passive state that neither contributes to nor interferes with the selection of visual input (Downing & Dodds, 2004; Olivers & Eimer, 2011).

The SIT model imposes a bottleneck between feature-based information in the attentional template and visual input during search. In brief displays, this predicts selection that is exclusive to a single target (Moore & Weissman, 2011, 2014). In longer displays, the bottleneck should slow search, because observers must switch the status of objects in VSTM to select and evaluate the presence of different targets. Recent behavioural and electrophysiological data, however, indicate pre-cues can modulate visual input for more than one target at the same time. Grubert and Eimer (2016) used event-related potentials (ERPs) to compare N2pc components to coloured targets that were preceded by coloured cues. On single-target trials, one of two pre-cued colours was presented in the search display. On dual-target trials, both pre-cued colours were presented in quick succession or simultaneously. The N2pc is thought to index the spatial selection of goal-relevant features in the scene (Cohen, Heitz, Schall, & Woodman, 2009; Eimer, 1996, 2015; Luck & Hillyard, 1994), and the data revealed comparable amplitudes and latencies for sequential and simultaneously presented colour targets preceded by one or two cues.

The electrophysiological results above are consistent with the parallel selection of visual input by an attentional template that encodes multiple target features (multiple-item template; MIT). Evidence to suggest independently cued features can also mediate perceptual categorisation was reported by Roper and Vecera (2012). They used a cued-RSVP task to elicit single- and dual-target searches for centrally presented objects in the presence of peripheral distractors. Distractors that matched the colour of one or two pre-cues decreased the accuracy of target detection; indicating attentional capture for different colours within a single frame (50 ms). Coloured distractors that matched neither cue had no effect on the accuracy of target detection, ruling out a stimulus-driven explanation for attentional capture in their task. Multiple colour cues have also been shown to mediate attentional capture during single-target search. Irons and colleagues contrasted RTs for targets that were presented at the same or a different location to a spatial cue that was red, green or blue. Prior to the task, red and green were specified as target colours and the data revealed spatial validity effects for targets at locations that were cued by either target colour (Irons, Folk, & Remington, 2012). This result is consistent with top-down modulation of visual input at the cued location for independently cued features. Notably, however, RTs tended to be faster for congruent compared to incongruent cue-target pairs, illustrating an asymmetry in the facilitation afforded by the two cued colours. Irons and colleagues interpreted this as evidence of a dissociation between selection and post-selection processes; with incongruent cue-target pairs disrupting decision processes or the consolidation of targets in VSTM, rather than the selection of task-relevant visual input (see also Adamo, Wozny, Pratt, & Ferber, 2010).

The results above suggest the selection of visual input for independently cued targets generalises from electrophysiological to behavioural responses during search. Evidence that MITs can also guide oculomotor behaviour has been reported by Beck and colleagues (Beck, Hollingworth, & Luck, 2012). They required observers to search multi-coloured arrays for an orientation-defined target. Single- and dual-target searches were elicited with one or two cues and observers were instructed to conduct sequential or simultaneous searches for both colours on dual-target trials. The data revealed sequences of fixations to objects of the same or alternate cued-colours proceeded at a similar rate, indicating observers could switch templates between saccades without incurring a delay. A comparison of the proportion of fixations to cued and uncued colours also revealed equivalent selectivity on single- and dual-target searches. Despite comparable accuracy, however, both fixation duration and manual responses were slower for dual- than single-target searches. Using a different method, Barrett and Zobay (2014) contrasted the speed and accuracy of single- and dual-target searches in free-view search displays. Their data revealed comparable slopes, but an increase in the intercepts of set size-by-RT functions for dual-target searches. SDT estimates fitted to the proportions of correct responses on target-present and -absent trials were also consistent with a small decrease in target discriminability when two targets were cued. The results of both studies indicate observers can use concurrently active features to guide search, but that the requirement to do so incurs costs in terms of the speed and accuracy of saccadic sampling and perceptual decisions.

The findings above are consistent with top-down selection of visual input by a multiple-item template during search. The distinction between ERP evidence for equivalent target-modulation and behavioural evidence of reductions in the speed and accuracy of target-detection, however, suggests the benefits of selection may not generalise to the mechanisms that support perceptual judgements and/or saccadic guidance during dual-target search. One possibility is that the single-item limit observed in previous studies reflects a constraint on the number of decision criteria that can be applied to selected objects during search. In this case, evidence for a single-item limit on attentional control would reflect a constraint on post-selection resources during dual-target search. Competition between selected objects based on similarity criteria for different cues may also disrupt the planning and execution of saccades during search (Godijn & Theeuwes, 2002). In this case, evidence for a single-item template may reflect the strategic prioritisation of a single-decision process during the programming of saccades to independently cued features (Cave et al., 2018; Grubert & Eimer, 2015; Rajsic, Ouslis, Wilson, & Pratt, 2017). Alternatively, dual-target costs in behavioural studies may reflect the distribution of post-selection resources across similarity distributions for separately cued targets (Barrett & Zobay, 2014; Menneer et al., 2009). In the absence of capacity-constraints, searching for one of two targets will increase decision-noise, because detection entails the comparison of visual input with different decision criteria. In this situation, decisions based on independent similarity distributions would increase the joint probability of distractors being confused with either target (Eckstein, 1998; Eckstein, Thomas, Palmer, & Shimozaki, 2000). The requirement to distribute resources to compare visual input against multiple cues may also reduce the accuracy of decision processes. Data from change-detection studies have shown that the precision of recall is inversely related to the number of remembered objects (Bays & Husain, 2008; Burmester & Wallis, 2012; Salmela & Saarinen, 2013), and recent evidence suggests this may reflect changes in the rate at which selected information is transferred into VSTM (Sewell, Lilburn, & Smith, 2014; Smith & Sewell, 2013). In dual-target search, selecting input associated with separately cued features may slow the transfer of selected information in VSTM, decreasing the fidelity of perceptual categorisation in brief displays (e.g. Irons et al., 2012) and slowing saccades during overt search (e.g. Beck et al., 2012).

The purpose of the current study was to investigate the nature of the dual-target cost during covert and overt search. Experiment 1 investigated the accuracy of perceptual judgements in brief displays that precluded eye movements on single- and dual-target searches. Experiments 2 investigated the accuracy of initial saccadic targeting (Awh, Armstrong, & Moore, 2006) on single- and dual-target searches. In the following experiments, we generalise the SDT models used by Houtkamp and Roelfsema (2009) to assess whether changes in target discriminability on dual-target searches are consistent with those predicted by (i) a single-item template limit on selection during search or (ii) multiple-item templates when two objects are cued. In the former, the dual-target cost is modelled by restricting target-present or -absent decisions to a single-item attentional template on dual-target searches (i.e., one cued orientation). In the latter, the dual-target cost is modelled as a function of the product of set size and the number of templates guiding search (i.e., 1 or 2 * set size on single- and dual-target searches, respectively). In addition, we derive noise- and capacity-limited versions of the single- and multiple-item template models to distinguish dual-target costs associated with decision-noise from those associated with a decrease in target discriminability when observers search for one of two cued targets (Barrett & Zobay, 2014; Houtkamp & Roelfsema, 2009; Smith, Lilburn, Corbett, Sewell, & Kyllingsbæk, 2016). Crossing single- and multiple-item templates with noise- and capacity-limited derivations produces four models, which are used to evaluate the contributions of decision-noise and capacity constraints on the dual-target costs predicted by distinct accounts of attentional control during dual-target search. To our knowledge, this is the first study to contrast the predictions of different explanatory models of the dual-target cost on the accuracy of perceptual decisions during covert search and saccadic targeting in free-view displays.

## Experiment 1

### Method

#### Observers

We used a small-*n* design to evaluate the descriptive and explanatory adequacy of SDT models of search. Our focus, therefore, is on the relationship between the observed and estimated data at individual and group levels of analyses. To equate sample size with relevant EEG studies (i.e., Eimer & Grubert, 2014; Grubert & Eimer, 2015) we recruited 12 observers. Data from one participant were excluded because they withdrew before completing the study. Of the remaining sample, seven were male and their ages ranged from 20 to 46 years (*M*_age_ = 23.20, *SD* = 4.96). All reported normal or corrected-to-normal visual acuity. Recruitment, consent and all experimental procedures conformed to American Psychological Association (APA) ethics standards.

#### Apparatus

The experiment was run on an IBM PC with a 19-in. CRT View Sonic G90fB monitor (Walnut, CA, USA). The display resolution was 1,240 × 768 pixels and the frame rate was 85 Hz. Stimulus presentation and data collection were controlled using custom-built software in MATLAB (Mathworks, Natick, MA, USA) with Psychophysics toolbox extensions (Brainard & Vision, 1997; Kleiner et al., 2007). Viewing distance was maintained at 57 cm using a fixed chin rest and responses were collected using a Cedrus RB-350 Response Pad (San Pedro, CA, USA). The experiment was conducted in a quiet, dimly lit room.

#### Stimuli

Displays contained red (*x* = 0.590, *y* = 0.326, *Y* = 4.54 cd/m^2^) Landholt’s *C*-shapes and grey (*x* = 0.281, *y* = 0.303, *Y* = 4.54 cd/m^2^) annuli that subtended 3.0° × 3.0°. Stimuli were presented at 12 equally spaced locations on the circumference of a virtual circle with a radius of 7.0°. *C*-shapes and annuli were presented on a uniform black (*x* = 0.590, *y* = 0.326, *Y* = 0.55 cd/m^2^) background.

#### Procedure

**Fig. 1:**
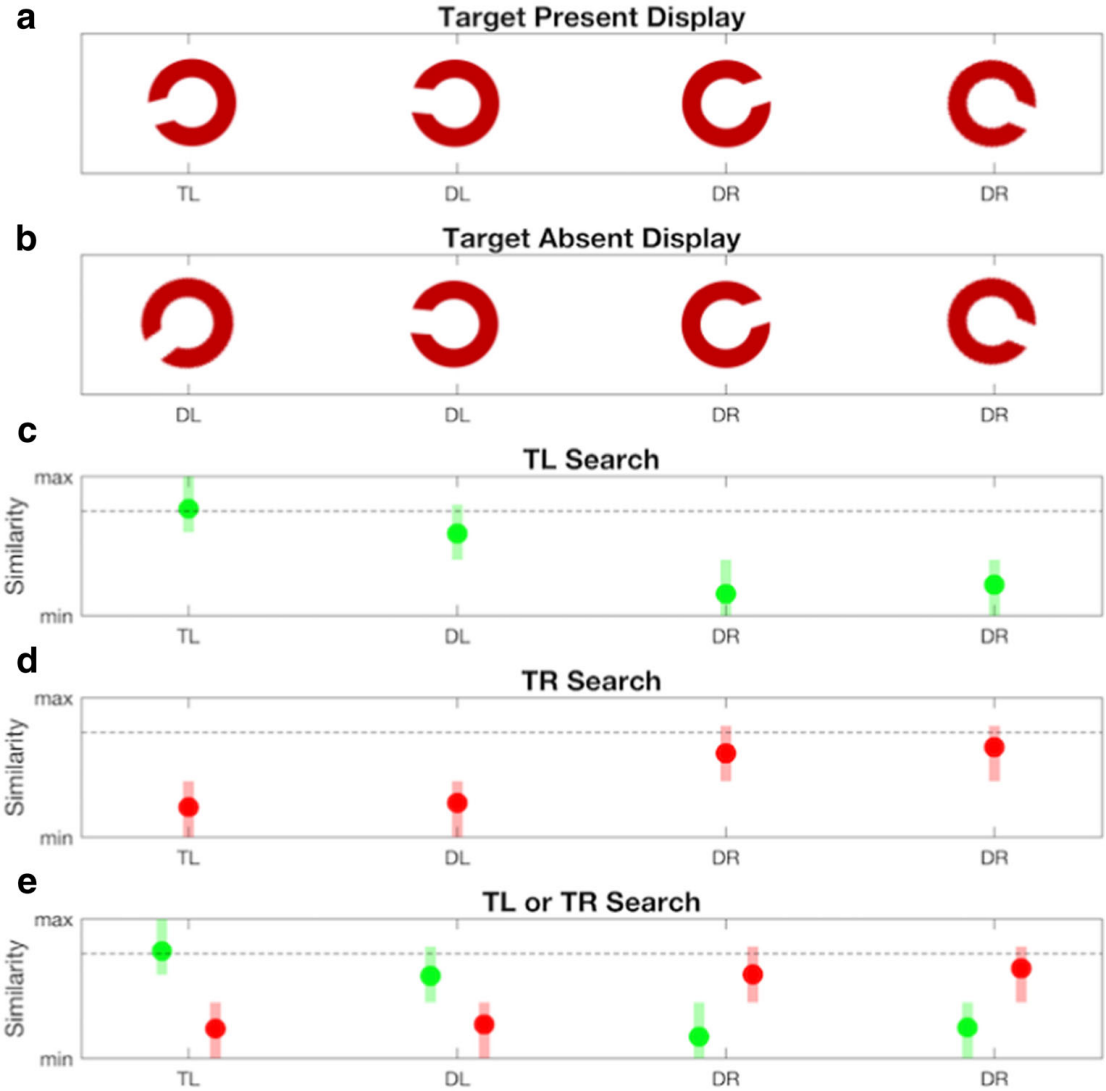
(**a**) Illustration of the orientation values assigned to *C*-shapes in a search display containing a leftward-leaning (TL) target. (**b**) Illustration of the orientation values assigned to *C*-shapes on a target-absent display. Distractors with similar and dissimilar orientations to TL are labelled DL and DR, respectively. (**c**) Hypothetical similarity values (filled circles) drawn from a normal distribution of internal responses (rectangles) to the *C*-shapes in panel A during a single-target search for TL. (**d**) Hypothetical similarity values and internal responses to the *C*-shapes in panel B during search for an absent rightward-leaning target. (**e**) Hypothetical similarity values and internal responses to the *C*-shapes in panel A during a dual-target search where observers are cued to search for a rightward- or leftward-leaning target. Dotted horizontal lines depict response criteria for target-present responses (similarity > criteria = target)

The experiment used a factorial design to manipulate four independent variables: Search type (single- or dual-target); trial type (target-present or -absent); target identity (Target-Left or -Right), and set size (1, 2 or 4 *C*-shapes). Experimental blocks contained four repetitions of this structure (96 trials). On each block of trials, two target and four distractor orientation values were assigned. Target orientations were sampled from 90° and 270° ± 15° to 30°. Distractor orientations for each observer differed from Target-Left or Target-Right by an angle of rotation that produced 80% accuracy on a pre-test (see below). Target and distractor values were fixed within experimental blocks to produce symmetric target-distractor similarity distributions for two targets among numerically equivalent subsets of leftward and rightward *C*s. (i.e., ± ~180°, see Fig. 1). This symmetry was designed to control potential differences in the perceptual salience of cued objects that might bias observers towards a serial strategy favouring one target over the other. The use of leftward and rightward *C*-Shapes was also designed to elicit competing similarity values between independently cued targets and numerically identical subsets of objects on dual-target searches. Displays could contain 1, 2 or 4 *C*-shapes, with the remaining locations occupied by an annulus to control for differences in perceptual crowding across set size.

**Fig. 2:**
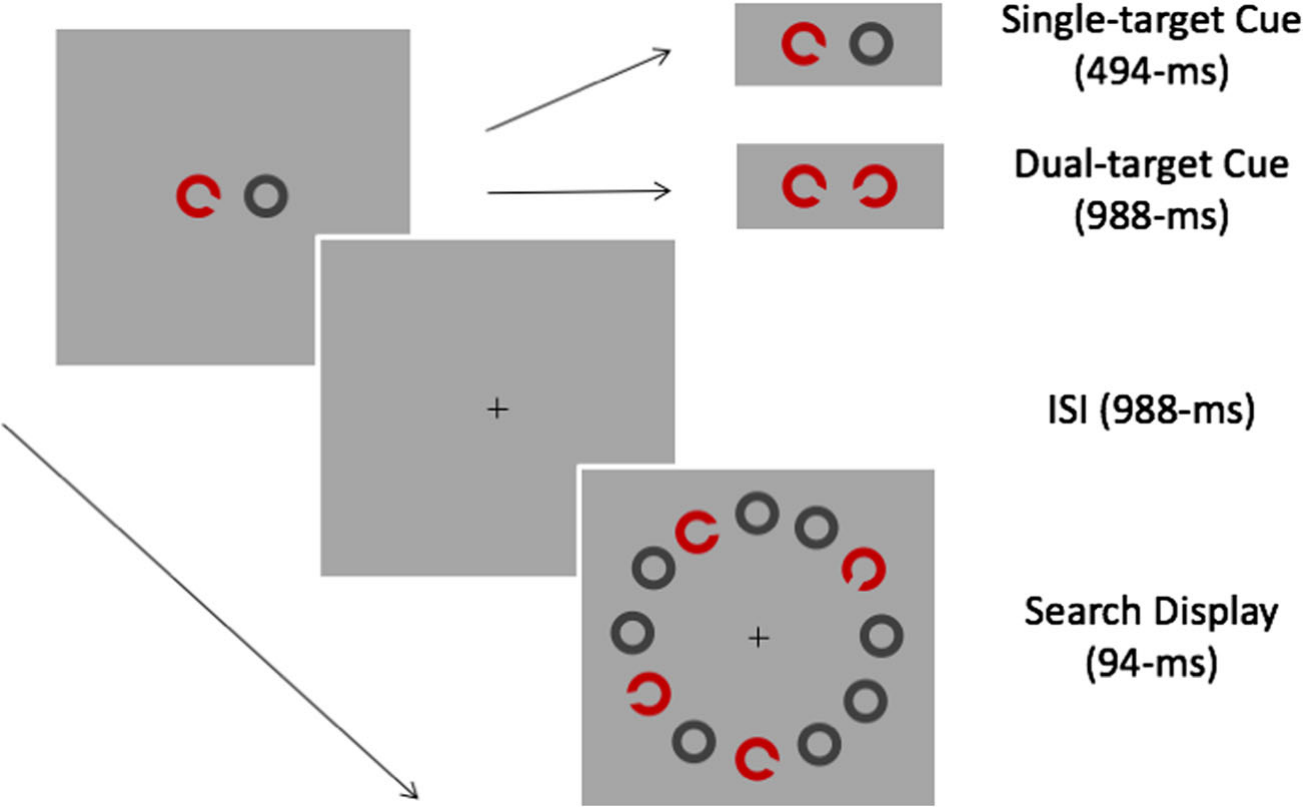
Sequence of events on each trial of the single-fixation search task. The search display contains the cued target (bottom) as well as a similar (upper left) and two dissimilar distractors

Figure 2 illustrates the sequence of events on each trial. Trials began with a fixation cross at the centre of the screen. After 500 ms, the fixation was replaced by a cue containing two objects centred on the horizontal midline ± 3° from fixation. Cues signalled the orientation of the subsequent search-target. On single-target trials, the cue consisted of an annulus and *C*-shape at the orientation assigned to Target-Left *or* Target-Right. On dual-target trials, the cue contained two *C*-shapes – one at the orientation assigned to Target-Left and one at the orientation assigned to Target-Right. To equate inspection times, single- and dual-target cues were presented for 494 ms and 988 ms, respectively. Cues were followed by a 988-ms blank inter-stimulus-interval before the onset of the search display. At a set size of four, target-absent displays contained one *C*-shape at the orientation assigned to each of the distractors (i.e., TL:R ± ∆°). At set size two, one leftward and one rightward distractor were randomly sampled from the four alternatives. At set size one, a single leftward or rightward distractor was sampled with equal probability. On target-present trials, displays contained one *C*-shape at the orientation assigned to Target-Left *or* Target-Right. Targets always replaced a similar distractor from the same (leftward or rightward) group and displays always contained one *C*-shape from a group that was cued. Search displays were presented for 94 ms and followed by blank screen, which remained visible until a response was recorded. Short-duration displays were used to equate processing time and prevent eye movements on single- and dual-target searches.

Observers completed ten blocks of trials in a single experimental session. In each block, single- and dual-target cues were equally likely for leftward and rightward targets. A cued target appeared in the search display on 50% of trials, and the order of presentation for each target, search type and trial was randomly assigned for each block. Prior to experimental sessions, observers completed two pre-test blocks of 80 trials. These presented a single *C*-shape in the same displays as the experimental session but varied the orientations of the target and distractor across the range ± 5° to 30° using a method of constant stimulus. Individual responses were fitted with a cumulative Gaussian function to estimate the angle of rotation required by each observer to distinguish targets from distractors on 80% of trials.

#### SDT models of search

**Fig. 3:**
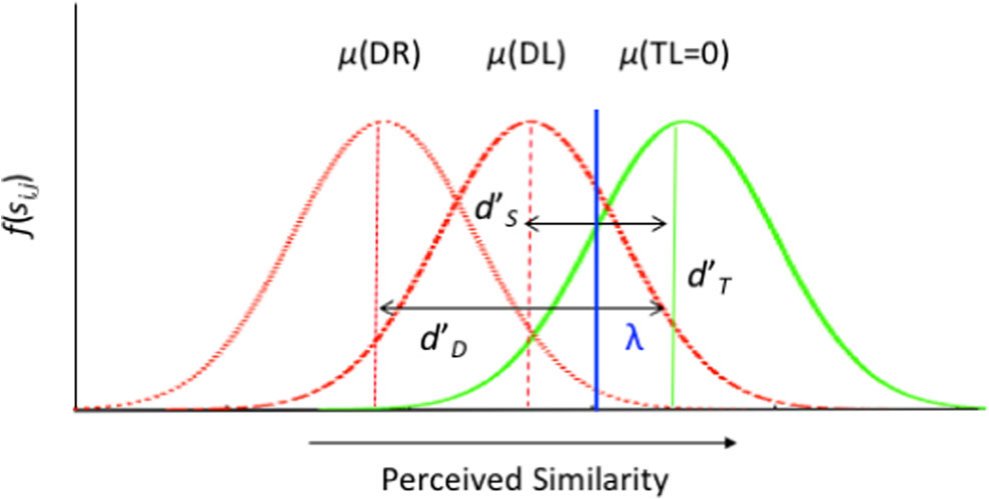
Illustrates the SDT model applied to a single-target search for a leftward leaning target (TL). The rightmost distribution represents the probability distribution elicited by the target. The centre and leftmost distributions represent the probability distributions elicited by the leftward (DL) and rightward distractors (DR). The target-distribution is centred at zero, and DL and DR distributions are centred at *μ*(DL) and *μ*(DR), respectively. Target discriminability is modelled in terms of the difference (*d*’) between the means of the distribution elicited by the target and those for similar (i.e., *d’*_*S*_ = *μ*(DL) – *μ*(TL)) and dissimilar (i.e., *d’*_*D*_ = *μ*(DR) – *μ*(TL)) distractors, respectively. The observer responds target present if the perceived similarity of any comparison exceeds the response criterion (*λ*)

The observer’s task on each trial is to classify the display in terms of the presence or absence of a target (‘yes-no’ decision). The statistical modelling of this process is based on signal detection theory (SDT) and the assumption of independent comparisons (Palmer, Verghese, & Pavel, 2000; Shaw, 1982). To classify the display, the observer independently compares the internal representation of orientation for each cue *i* to the visual response to each object in the display *j*. The result of each comparison describes the perceived similarity between the cue and the object, which is represented by a real number *s*_*ij*_. To classify the display, observers evaluate whether the maximum similarity value is less than or equal to a response criterion. The cumulative probability distribution function *P*(*s*_*ij*_ ≤*R*) ;of s_*ij*_ depends on three factors: (1) The type of comparison *C*(*i,j*), which can be a target(*t*) similar distractor(*s*) or dissimilar distractor(*d*), depending on the relationship between the cue and the object in the display. (2) The set size *D*, which can equal 1, 2 or 4. (3) The number *T* of templates used to guide search, which is determined by the number of cues. These dependencies are indicated by the notation *P*(*s*_*ij*_ ≤ *R* | *C,D,T*). As a simplifying assumption, all comparisons between cues and objects with opposite orientations are modelled by the same probability distribution. In particular, the target is considered a dissimilar distractor for the second cue on dual-target searches. In agreement with standard SDT (Green & Swets, 1974; Wickens, 2002), probability distributions are modelled as Gaussian with mean *μ*(*C,D,T*) and variance 1, i.e., *P*(*s*_*ij*_ ≤ *R* | *C,D,T*) = Φ (*R* – *μ*(*C,D,T*)), where Φ denotes the standard normal cumulative probability distribution (Fig 3).

To distinguish between theoretical accounts of attentional control during search, we introduce additional assumptions to derive two types of SDT model. Multiple-item template (MIT) models simulate display classification based on the evaluation of similarity values for independently cued feature values during dual-target search. We assume that the observer reports target-absent if none of the comparisons exceed a response criterion *λ*(*D,T*). Because of independence, the probability that none of the comparisons exceed the response criterion equals the product of the probabilities for each individual comparison being less than *λ*(*D,T*):

1 $$ P\left(\mathrm{target}-\mathrm{absent}\right)={\prod}_{\left\{i,j\right\}}P\left({s}_{ij}\le \lambda \left(D,T\right)|C\left(i,j\right),D,T\right) $$

In Eq. 1, the product is over all pairwise comparisons between cues and objects at a given set size and type of search (*D,T*). Conditional probabilities for hits and false alarms are obtained as 1*-P*(target-absent), depending on whether the target is present or absent in the display.

Applying Eq. 1 directly to the observed data would require the estimation of six decision thresholds *λ*(*D,T*) and 18 *μ*(*C,D,T*) parameters (i.e., separate decision thresholds and *μ* estimates for single- and dual-target searches by comparison type and set size). However, the experimental design yields only 12 observations for each subject (i.e., the hit and false-alarm rates in each of the search types by set size conditions). To derive useful and testable models, we therefore impose restrictions on the model parameters: First, we note that model predictions do not change if the same constant value is added to all parameters appearing together in a product term (1). To fix the absolute scale, we therefore set *μ*(*C*=*t,D,T*) = 0 (i.e., the target distribution is centred at zero). Target discriminability is modelled as the difference between the means of the distributions for the target and those for similar and dissimilar distractors. For similar distractors, *d'*_*S*_(*D,T*) = *μ*(*C*=*s,D,T*)-*μ*(*C*=*t,D,T*). For dissimilar distractors, *d'*_*D*_(*D,T*) = *μ*(*C*=*d,D,T*)-*μ*(*C*=*t,D,T*). We then distinguish between noise- and capacity-limited search. In the former, we assume that *d*’ is independent of set size and search type, i.e., *d*’(*C,D,T*) = *d*’(*C*). As a shorthand, we set *d*’(*C*=*s*) = *d*’_*S*_ and *d*’(*C*=*d*) = *d*’_*D*_. For capacity-limited models, we assume that *d’* scales with the inverse root of the total number of comparisons in each search: with *d’*(*C*=*t*)=0, this can be written as *d*’(*C*=*s*, *D, T*) = *d*’_*S*_/√(D*T) with *d*’_*S*_ =*d’*(*C*=*s*, *D*=1,*T*=1). This parameterisation produces negative *d’* values for distractors that are differentiated from the target distribution on the basis of their dissimilarity from the cue, while scaling *d’* by 1/√(D*T) conforms to the decline in discriminability predicted by the SDT sample-size model of search (see Corbett & Smith, 2017; Palmer, 1994; Smith & Sewell, 2013). For MIT models, we also make an equivalence assumption between searches that entail the same number of comparisons. This implies that single-target searches for *D*=2 or 4 are equivalent to dual-target searches for *D*=1 or 2, respectively. For *d*’, this assumption is consistent with both noise- and capacity-limited models. For *λ*, we set *λ*(*D,T*) = *λ*(D*T), as the number of comparisons is the product of set size and cued orientations. The various *λ* values are denoted *λ*_1_, *λ*_2_, *λ*_4_, *λ*_8_. Equation 1 provides a general form that can be applied to estimate the proportion of hits and false alarms across each search type by set size condition. For example, the estimated hit rate for the noise-limited MIT model when Target-Left is present at set size 4 is given by

2 $$ {P}_{MIT}\left(\mathrm{Hit}|D=4,T=2\right)=1-\varPhi \left({\lambda}_8\right)\varPhi {\left({\lambda}_8-{d}_S^{\prime}\right)}^3\varPhi {\left({\lambda}_8-{d}_D^{\prime}\right)}^4 $$

as there is one cue-target comparison (CL-TL), three comparisons with similar distractors (CL-DL & CR-DR * 2) and four comparisons with dissimilar distractors (CR-TL, CR-DL & CL-DR * 2).

In contrast to MIT models, single-item template (SIT) models simulate search guided by a single attentional template. On a dual-target search, target detection is based on pairwise comparisons between the active attentional template and the objects in the display. We assume that the observer reports target absent if these comparisons fall below a response criterion *λ*(*D,T*). To represent the qualitative distinction between cues during the comparison process, SIT derivations contain separate terms to compute similarity values for objects depending on which template is active during search. For example, the estimated hit rate for the noise-limited SIT model on a dual-target search with a set size of 4 is given by

3 $$ {P}_{SIT}\left(\mathrm{Hit}|D=4,T=2\right)=1-0.5\ast \left(\varPhi \left({\lambda}_4\right)\varPhi \left({\lambda}_4-{d}_S^{\prime}\right)\varPhi {\left({\lambda}_4-{d}_D^{\prime}\right)}^2+\varPhi {\left({\lambda}_4-{d}_S^{\prime}\right)}^2\varPhi {\left({\lambda}_4-{d}_D^{\prime}\right)}^2\right) $$

In this equation, the observer randomly selects one of the cues and performs a single-target search based on the selected (active) attentional template. If the selected template matches the target in the display, performance will be equivalent to a single-target search. If the selected template matches the target that does not appear, hits are highly unlikely, because the cue-target comparison will yield a low similarity value. Accuracy across dual-target searches is predicted to be the average of the two terms. As for MIT models, we also distinguish noise- and capacity-limited derivations of SIT search. For the former, we assume *d*’ is independent of set size, i.e., *d*’(*C,D,T*) = *d*’(*C*). For the capacity-limited model, *d’* scales with the inverse root of the number of objects in the display, because the number of active attentional templates is fixed at 1. With *d’*(*C*=*t*)=0, this can be written as *d*’(*C*=*s*,*D,T*) = *d*’_*S*_/√*D* with *d*’_*S*_ =*d’*(*C*=*s*, *D*=1,*T*=1). Similarly, we set *λ*(*D,T*) = *λ*(*D*) to model decision-noise and changes in threshold as a function of the number of comparisons between one attentional template and objects in the display. The various *λ* values are denoted *λ*_1_, *λ*_2_, *λ*_4_.

The MIT and SIT models above simulate distinct strategies of attentional control during dual-target search. For MIT models, accuracy on single- and dual-target searches is predicted by an inverse relationship between the product of set size and the number of orientations cued prior to search. For SIT models, accuracy on dual-target searches is predicted by a comparison process that is restricted to a single cued orientation during search (Houtkamp & Roelfsema, 2009). For MIT and SIT models, noise-limited derivations capture an important general property of SDT, which is the predicted association between the probability of false alarms and the number of comparisons required to classify the display: Increasing the number of comparisons during search has a multiplicative impact on the likelihood of mistaking a distractor for a target (Eckstein, 2011; Verghese, 2001; Wilken & Ma, 2004). Capacity-limited derivations of MIT and SIT assume an additional dependency between target discriminability and the number of comparisons required to classify the display. Comparing noise- and capacity-limited versions of the MIT and SIT models provides a means to distinguish the contribution of decision-noise from changes in sensitivity during single- and dual-target searches (Palmer, 1994; Smith & Sewell, 2013). To contrast these predictions, SIT and MIT, noise- and capacity-limited models were fitted to each observer’s data. This required the estimation of five or six parameters (two *d*’ and three or four λ values) producing 5 or 6 error degrees of freedom. Fitting was carried out using maximum likelihood estimation and goodness of fit was assessed using a parametric bootstrap technique for Pearson’s χ^2^ statistic. To do this, SDT models were fitted to each observer’s data to compute the observed χ^2^ statistic. Parameter estimates for the fitted model were then used to simulate observations over *K* repetitions of the experiment (we used *K* = 500). By re-fitting and computing the χ^2^ statistic for each repetition, the χ^2^ distribution that would derive under the fitted model was estimated. Comparing the observed χ^2^ statistic against this χ^2^ distribution yielded the probability (*p*-value) that the fitted model generates data that are at least as extreme as those observed. The larger this probability, the ‘more typical’ the observed data are under the fitted model, and the stronger the evidence for its plausibility. As an additional, more intuitive measure of fit, we also calculated the mean absolute difference between corresponding observed and estimated probabilities. For brevity, we report mean group estimates in the text. Model estimates for individuals are reported in the Electronic Supplementary Material (ESM), where the mean absolute difference between observed and estimated values is labelled as ‘Fit 2’.

### Results

#### Orientation thresholds

Best-fitting Gaussian probability density functions for each observer yielded a mean 80% accuracy threshold of ± 19.72° (SD = 3.1°) at a set size of one.

#### Accuracy

Accuracy for leftward and rightward leaning targets did not differ significantly (mean 0.703 vs. 0.722; *t*(10) = 1.31, *p* > 0.20, Cohen’s *d* = 0.34), and the remaining analyses collapse responses across both targets during search. Table 1 presents the mean proportion of hits, false alarms and correct responses by set size for single- and dual-target searches. As expected, accuracy was lower on dual- than single-target searches. A 2 × 3 repeated-measures ANOVA on proportion-correct data yielded significant main effects of search type, *F*(1,10) = 144.80, *p* < 0.001 , *η*_p_^2^ = .94, set size, *F*(2,20) = 138.80, *p* < 0.001, *η*_p_^2^ = .93, and a significant Search Type by Set Size interaction, *F*(2,20) = 4.97, *p* = 0.018, *η*_p_^2^ = 0.32. Two-template SDT models predict a reciprocal relationship between search accuracy and the number of comparisons required to classify the display. Planned contrasts to compare single- and dual-target searches across equivalent set sizes revealed non-significant differences at two, *t*(10) = .14, *p* = 0.89, *d* = 0.04, and four comparisons, *t*(10) = .67, *p* = 0.52, Cohen’s *d* = 0.21, respectively. These data provide initial evidence that accuracy is related to the number of comparisons required to classify the display rather than qualitatively different strategies of attentional control on single- and dual-target searches.

**Table 1:** Mean proportion of hits [P(H)], false alarms [P(F)] and correct responses [P(C) = (P(H) + 1-P(F))/2] by Search Type and Set Size in Experiment 1

	Single target			Dual target		
Set size	1	2	4	1	2	4
P(H)	.94 (.02)	.80 (.03)	.64 (.03)	.80 (.03)	.63 (.03)	.55 (.05)
P(F)	.24 (.04)	.24 (.03)	.35 (.03)	.22 (.04)	.32 (.03)	.45 (.05)
P(C)	.85 (.02)	.78 (.02)	.65 (.02)	.79 (.02)	.66 (.02)	.55 (.01)

Standard errors in parenthesis

#### Model estimates and fits

**Fig. 4:**
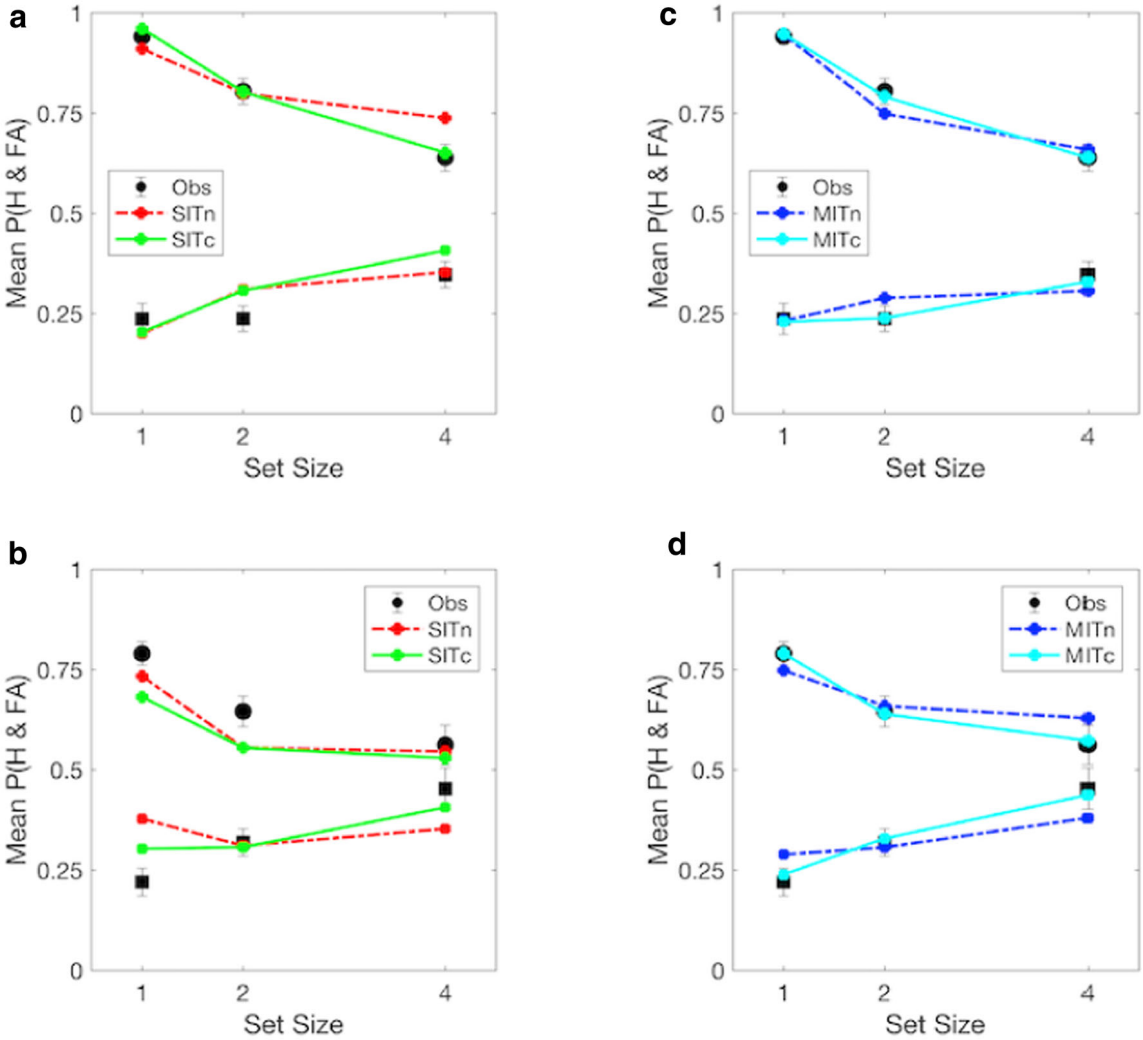
Mean proportion of observed and estimated hits (circles) and false alarms (squares) by set size and search type. Single-item template, noise- (SITn) and capacity-limited (SITc) estimates on single- (**a**) and dual-target (**b**) searches. Multiple-item template, noise- (MITn) and capacity-limited (MITc) estimates on single- (**c**) and dual-target searches (**d**). Error bars represent standard errors of observed means

Figure 4 plots the mean observed against the mean predicted proportions of hits and false alarms for each SDT model by search type and set size. Panels A and B reveal predicted values for the SIT models that diverge considerably from the observed data. Panels C and D, in contrast, indicate much better fits between predicted and oberved data, with the MIT, capacity-limited model producing mean predicted values within 1 standard error of the observed means for single- and dual-target searches across set sizes. At set size 4, predicted hit and false-alarm rates for the MIT, noise-limited model fall outside the standard error of observed values on single- and dual-target searches, indicating a worse fit than for the MIT, capacity-limited model. Table 2 presents mean parameter estimates of target-disriminability (*d’*) and response bias (*λ*) across equivalent set sizes, as well as statistical comparisons for the four models. All reveal a monotonic increase in *λ* as a function of set size. Estimates of *d*’_*D*_ were smaller than those for *d*’_*S*_ for the both SIT models and the MIT, noise-limited model, reversing the expected pattern of discriminability for targets among similar and dissimilar distractors. Allowing *d*’s to scale with set size produced less variable *λ* estimates, and the expected relationship between *d*’_*S*_, and *d*’ _*D*_ for the MIT model only. AIC values were also smaller for the MIT than SIT models, with the smallest value obtained under the MIT, capacity-limited model – indicating a better fit between the observed and predicted hits and false alarms when accuracy was inversely scaled by the product of set size and the number of cued orientations. This group-level advantage was replicated at the individual level, where AIC values were smallest and goodness-of-fit indices largest for the MIT, capacity-limited model for 10/11 observers. The mean absolute difference between observed and estimated data was also smaller for the MIT, capacity- (0.03) than the MIT, noise-limited (0.05), and SIT, capacity- (0.07) and noise-limited (0.06) models (see Tables S1a to S1d in the ESM for individual estimates and fit-indices).

**Table 2:** Mean (standard error) parameter estimates for the SIT and MIT noise- (n) and capacity-limited (c) models across equivalent set sizes in Experiment 1

	*λ* by search and number of comparisons							
	ST1	ST2-DT1	ST4-DT2	DT4	*d*’_*S*_	*d’*_*D*_	Fit	∑ AIC
SITn	-1.43 (.11)	-0.64 (.09)	-0.30 (.09)	-	-2.34 (.19)	-1.21 (.10)	< 0.001	1172.37
SITc	-2.05 (.19)	-1.56 (.17)	-1.36 (.18)	-	-2.94 (.25)	-2.33 (.17)	0.05 (.02)	1052.98
MITn	-1.94 (.25)	-.042 (.09)	-0.22 (.08)	0.22 (.11)	-2.72 (.29)	-1.05 (.11)	0.09 (.07)	932.84
MITc	-1.86 (.18)	-1.55 (.16)	-1.39 (.17)	-1.20 (.20)	-2.65 (.23)	-3.64 (.46)	0.38 (.10)	800.28

SIT models assume the number of comparisons is equal to display set size. On dual-target trials, DT4 = 8 comparisons. Subscripts S and D denote *d*’ estimates for distractors with orientations similar and dissimilar to the target appearing in the display

*AIC* Akaike Information Criterion

### Discussion

The results above are consistent with previous demonstrations of the dual-target cost; observers were significantly less accurate on dual- compared to single-target searches across set sizes 1, 2 and 4. Our data extend previous findings from free-view (i.e., Barrett & Zobay, 2014; Menneer et al., 2007) to brief displays that preclude eye movements during single- and dual-target search. To determine whether the reduction in accuracy is consistent with comparisons based on one or two cued-orientations, we contrasted the observed probability distributions of hits and false alarms with those predicted by SIT and MIT noise- and capacity-limited SDT models of search. These revealed poor fits for the SIT models; indicating accuracy exceeding that predicted by a single-item limit on selection or decision criteria during search. In contrast, both MIT models provide a good approximation of observed accuracy distributions on single- and dual-target searches (see Fig. 4), with the range of estimated parameters and statistical comparisons favouring the capacity- over the noise-limited model at both individual and group-level analyses. The close fit between the observed and predicted data for the MIT capacity-limited model indicates the dual-target cost is (1) best approximated by an inverse relationship between accuracy and the product of set size and the number of cued orientations and (2) exceeds that predicted by an increase in decision-noise alone during independent searches for each cued-target. This result suggests the distinction between equivalent electrophysiological responses for independently cued targets (e.g. Grubert & Eimer, 2016) and behavioural indices of the dual-target cost (e.g. Irons et al., 2012) is attributable to increases in decision-noise as well as the distribution of capacity-limited decision resources when observers evaluate the presence of more than one potential target during search.

SDT models of single-target search have been successfully used to characterise the relationships between decision-noise, discriminability and set size for different stimuli (e.g. Eckstein et al., 2000; Põder, 2017; Shaw, 1980; Smith, 2010; Verghese, 2001). Our analyses suggest extending set size to quantify the number of comparisons between items in VSTM (i.e., memory set size) and objects in the display is sufficient to explain the dual-target cost in brief displays. In information theoretic terms, capacity-limited models consider display duration a limit on the accumulation of evidence during search, which is divided by the number of objects in the display (sample-size model). Our data suggest the limit on accumulation can be described by scaling *d*’ by the square root of the number of comparisons required to classify the display (i.e., *d*’/√(attentional templates * set size)). This interpretation is consistent with capacity-limited decision processes based on the concurrent evaluation of similarity distributions for independently cued targets.

Previous research suggests perceptual decisions and eye movements are both informed by an evaluation of the maximum sensory evidence of the target (Beutter, Eckstein, & Stone, 2003; Najemnik & Geisler, 2008). Other findings, however, have highlighted the parallel nature of saccadic programming and the potential for competition between neurons coding the locations of different stimuli during the planning and execution of eye movements (McPeek & Keller, 2001; Theeuwes, Kramer, Hahn, & Irwin, 1998). Saccadic latencies to cued-targets are slowed by the presentation of a distractor with cued features (Ludwig & Gilchrist, 2003), and presenting two cued targets during saccades elicits near-equivalent proportions of fixations to each target (Beck & Hollingworth, 2017). Shifts of attention during presaccadic planning are also thought to entail VSTM resources during the retention and evaluation of information used to evaluate postsaccadic visual input (Deubel & Schneider, 1996). The foveation of likely targets also requires the translation of similarity values from a parallel to a graded-serial representation during the planning and execution of saccades. Applied to dual-target search, these findings suggest competition between similarity maxima for independently cued features may disrupt processes that are specific to saccadic targeting during search. Experiment 2 was designed to establish whether the dual-target cost in saccadic targeting is consistent with that predicted by (1) a single-item limit on selection or (2) selection based on a multiple-item template during search for one of two potential targets.

## Experiment 2

### Method

#### Observers

Twelve observers were recruited to the study: Two were male and age ranged from 18 to 52 years (*M*_age_ = 22.70, *SD* = 1.81). All reported normal or normal-to-corrected visual acuity.

#### Apparatus

The experiment was run on an IBM PC with a 21-in. HP Trinitron P1130 monitor CRT monitor (Walnut, CA, USA). The display resolution was 1,280 × 1,024 pixels and the frame rate 100 Hz. Stimulus presentation and data collection were controlled using custom-built software in MATLAB (Mathworks, Natick, MA, USA) with Psychophysics toolbox extensions (Brainard & Vision, 1997; Kleiner et al., 2007). Viewing distance was maintained at 57 cm using a fixed chin rest and eye movements were recorded with an EyeLink 1000 video-based eye tracker (SR Research Ltd., Ottawa, ON, Canada) at a sampling rate of 1,000 Hz and spatial resolution of < 0.02°.

#### Stimuli

Displays contained four red (*x* = 0.566, *y* = 0.333, *Y* = 5.49 cd/m^2^) Landholt’s *C*s presented on a black background (*x* = 0.294, *y* = 0.283, *Y* = 0.82 cd/m^2^). *C*s subtended 5.0° × 5.0° at equally spaced locations on the circumference of a virtual circle with a radius of 7.0°. In contrast to Experiment 1, stimuli were designed to be large enough to discriminate leftward and rightward orientation from the central fixation and placed at more eccentric locations that required orthogonal saccadic vectors. As set size was fixed at four objects, the ellipses used to control crowding across different set sizes in Experiment 1 were not included in displays.

#### Procedure

The experiment used a factorial design to manipulate two independent variables: Search type (single- or dual-target) and stimulus type (target, similar and dissimilar distractor). On each block, Target-Left and Target-Right orientations were sampled from 90° and 270° ± 15° to 30°, respectively. Distractor orientations were assigned as Target-Left and -Right ± 25°. Target and distractor orientations were fixed and the order of presentation for each target and search type were randomly assigned within each block.

**Fig. 5:**
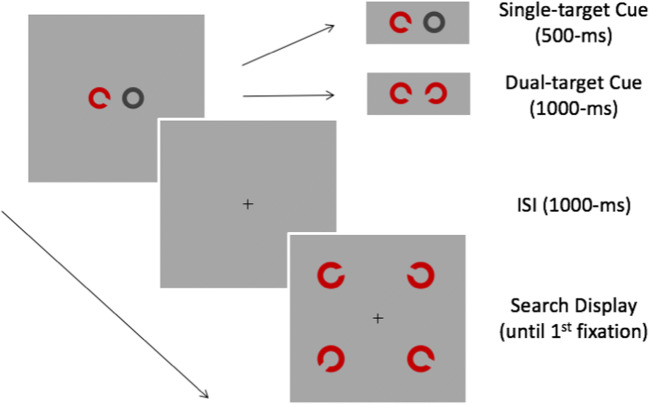
Sequence of events on each trial of the overt search task. In this display, the target and similar distractor are in the bottom-right and top-left corners, respectively

Figure 5 illustrates the sequence of events on each trial. Trials began with a fixation cross at the centre of the screen. After 500 ms, the fixation was replaced by a cue containing two objects on the horizontal midline at ± 3.5° from the centre of the screen. On single-target searches, the cue consisted of one *C*-shape at the orientation assigned to Target-Left *or* Target-Right and one annulus. On dual-target searches the cue contained two *C*-shapes at the orientations assigned to Target-Left *and* Target-Right. Single- and dual-target cues were presented for 500 ms and 1,000 ms, respectively, before being replaced by a fixation cross for 1,000 ms. Search displays followed and always contained four objects – a cued target and one similar and two dissimilar distractors – which were randomly assigned to the four possible locations. Observers were instructed to fixate the object that matched a cue, and responses were measured using a box criterion to record the first fixation falling within the 5.0° × 5.0° area surrounding each *C*-shape in the display. This method excludes saccades falling outside stimulus locations but produces results similar to the nearest endpoint criteria (Eckstein, Beutter, & Stone, 2001) and provides unambiguous indices of object selection during search. Once a response had been recorded, the bounding square surrounding the fixated object was coloured red for 50 ms and the search display was replaced by a blank screen for 500 ms.

Observers completed a short practice (20 trials) followed by four blocks of 60 trials in a single experimental session. Before each block, a 5-point calibration sequence was performed to ensure fixations to each *C*-shape could be recorded. Following successful calibration (gaze errors < 0.5°), observers were prompted to press a key to begin each block of trials.

#### SDT models of saccadic targeting

The observer’s task on each trial is to indicate which of four stimuli match the cue by fixating the target. Our statistical description of this task uses the SDT framework derived for Experiment 1. Following the standard approach to modelling forced-choice tasks (Green & Swets, 1974; Kingdom & Prins, 2010; Wickens, 2002), we assume that the subject compares cued objects (*i* = 1 to 2) with those in the display (*j* = 1 to 4) and fixates the comparison that produces the maximum similarity *s*_*ij*_ to select the most likely target. The probability of the cue-object comparison (*i,j*) to yield maximum similarity is given by

4 $$ P\left({s}_{i,j}\ \mathit{\max}\right)={\int}_{-\infty}^{\infty }p\left(y|C\left(i,j\right),D,T\right){\prod}_{\left(i\hbox{'},j\hbox{'}\right)\ne \left(i,j\right)}P\left({s}_{i,j}\le y\ |C\left({i}^{\prime },{j}^{\prime}\right),D,T\right)\  dy. $$

In Eq. 4, *p*(*y*|*C(i,j),D,T*) denotes the probability density associated with *P*(*s*_*ij*_ ≤ *y* | *C(i,j,),D,T*). The equation can be interpreted as follows: The product term in the integrand describes the probability that all comparisons different from (*i,j*) have results less than *y*. Weighing this value with the probability density for obtaining response *y* in the (*i,j*) comparison and integrating over all possible values of *y* yields the total probability for selecting comparison (*i,j*).

With *D* fixed to 4 and setting *μ*(*C*=*t,D,T*) = 0 as in Experiment 1, the model has four free parameters, which represent the perceived similarity of similar (*s*) and dissimilar (*d*) distractors on single- (ST) and dual-target (DT) searches; *d*’(*s*_ST_), *d*’(*d*_ST_), *d*’(*s*_DT_) and *d*’(*d*_DT_). For each observer, there are four observed quantities, i.e., the probabilities for selecting the target and similar distractor on single- and dual-target searches, respectively (the probability for choosing the dissimilar distractor is fixed because of *P*(*t*)+*P*(*s*)+2*P*(*d*) = 1). Without any further restrictions on the *d*’ parameters, the model is fully identified and would reproduce the observations perfectly. However, in analogy to Experiment 1, we consider noise-limited SIT and MIT models with *d*’(*s*_ST_) = *d*’(*s*_DT_) = *d*’_*S*_ and *d*’(*d*_ST_) = *d*’(*d*_DT_) = *d*’_*D*_. We also compute estimates for a capacity-limited MIT model in which *d*’ scales with the number of comparisons required to search for one or two cued-targets (i.e., *d*’(*s*_ST_) = *d*’_*S*_, *d*’(*s*_DT_) = *d*’_*S*_/√2 and *d*’(*d*_ST_) = *d*’(*d*_DT_) = *d*’_*D*_). As set size is fixed across search types in this experiment, scaling *d’*s by √2 models a capacity limit that is specific to the MIT model when VSTM resources are distributed across independent attentional templates during search. All three models have two free parameters and 2 error degrees of freedom at the subject level.

The subject does not directly report the chosen comparison (*i,j*) but only the corresponding display object *j*. Therefore, the statistical model has to provide expressions for *P*(*j*) rather than *P*(*i,j*). This does not cause any complications for single-target searches, as there is only one cue *i*=1 and *P*(*j*) = *P*(*i*=1,*j*). For dual-target searches in the MIT model, saccades to object *j* could be based on the comparison for either cue, i.e., (*i*=1,*j*) or (*i*=2,*j*) so that the corresponding probabilities have to be added: *P*(*j*) = *P*(*i*=1,*j*)+*P*(*i*=2,*j*). For the SIT model, the overall probability of fixating the target in a DT search is given by

5 $$ {\displaystyle \begin{array}{c}P(Target)=\frac{1}{2}\ \Big({\int}_{-\infty}^{\infty}\varphi (y)\varPhi \left(y-{d}_S^{\hbox{'}}\right){\varPhi}^2\left(y-{d}_D^{\hbox{'}}\right) dy\\ {}+{\int}_{-\infty}^{\infty}\varphi \left(y-{d}_D^{\hbox{'}}\right){\varPhi}^2\left(y-{d}_S^{\hbox{'}}\right)\varPhi \left(y-{d}_D^{\hbox{'}}\right) dy\Big).\end{array}} $$

In this equation, the first integral denotes the probability of target fixation when the observer activates the attentional template matching the target in the display. The second integral denotes the probability of target fixation when the observer activates the template matching the cued target that is not presented in the display. On these trials, the probability of fixating the target is low because the comparison between the attentional template and the target is unlikely to yield the maximum similarity (i.e., TL cue against TR target). For MIT models, the overall probability of selecting the target object in the noise-limited model is given by

6 $$ {\displaystyle \begin{array}{c}P(Target)={\int}_{-\infty}^{\infty}\varphi (y){\varPhi}^3\left(y-{d}_S^{\hbox{'}}\right){\varPhi}^4\left(y-{d}_D^{\hbox{'}}\right) dy\\ {}+{\int}_{-\infty}^{\infty}\varphi \left(y-{d}_D^{\hbox{'}}\right)\varPhi (y){\varPhi}^3\left(y-{d}_S^{\hbox{'}}\right){\varPhi}^3\left(y-{d}_D^{\hbox{'}}\right) dy.\end{array}} $$

This equation is interpreted as follows. On a dual-target search, the participant performs one target comparison, three similar-distractor comparisons and four dissimilar-distractor comparisons. The first integral describes the probability that the subject selects the comparison between the target cue and target object. The second pertains to choosing the comparison between the cued target that is not present and the target object, which is considered a dissimilar-distractor comparison (i.e., TL cue against TR target). Models are again fitted using maximum-likelihood estimation. For the computation of integrals, we make use of the fact that φ is a normal distribution and apply Gauss-Hermite quadrature (Press, Teukolsky, Vetterling, & Flannery, 2007). Mean estimates and overall goodness-of-fit statistics are reported in the text. Individual estimates and fit-statistics are provided in the ESM.

### Results

#### Accuracy

Trials in which fixations to a display object were recorded in less than 100 ms or longer than 3 standard deviations from the mean of single- and dual-target searches, respectively, were excluded from further analyses (*M* = 0.04). Accuracy for Target-Left and Target-Right were not significantly different, *t*(11) = 0.07, *p* > 0.95, Cohen’s *d* = 0.019, and the following analyses collapse responses across both targets. Table 3 lists the mean proportion of recorded first fixations and saccadic latencies to targets and distractors during single- and dual-target searches. Data for single-target searches reveal highly accurate saccadic guidance, with observers demonstrating a clear bias to fixate targets over distractors. Relative to the target presented in the display, observers were also more likely to fixate similar than dissimilar distractors, illustrating similarity-based competition for selection during search. On dual-target searches, the distribution of fixations to targets and distractors was more equal. Planned comparisons yielded a significant decrease in target-fixations, *t*(11) = 16.61, *p* < 0.001, Cohen’s *d* = 4.80, and increase in fixations to dissimilar distractors, *t*(11) = 12.32, *p* < 0.001, Cohen’s *d* = 3.56, on dual- compared to single-target searches. The proportion of fixations to similar distractors for each type of search was not significantly different, *t*(11) = 0.52, *p* > 0.05, Cohen’s *d* = 0.15.

**Table 3:** Mean proportion and median saccadic latencies (milliseconds) of first fixations to targets and distractors on single- and dual-target searches in Experiment 2. Distractors are labelled similar (DS) and dissimilar (DD) relative to the orientation of the cued-target presented in the display

	Single target			Dual target		
Object	T	DS	DD	T	DS	DD
P (fixation)	.71 (.04)	.18 (.03)	.05 (.01)	.47 (.04)	.16 (.02)	.19 (.01)
Mean SL	671 (.15)	617 (.19)	548 (.18)	647 (.14)	610 (.21)	651 (.15)

Standard errors of means are reported in parentheses

The proportion shown for dissimilar distractors (DDs) is half the proportion of fixations made to both dissimilar distractors in the display

#### Saccadic latencies

Our focus in this study was to investigate changes in the accuracy of saccadic targeting during single- and dual-target searches. In Experiment 1, short-duration displays constrained the accumulation of information equally for both types of search. In the saccadic tasks, however, observers may compensate for changes in the fidelity of guidance by sampling information longer before initiating saccades. In order to assess potential speed-accuracy trade-offs, we computed median saccadic latencies to targets and distractors for single- and dual-target searches for each observer (lower row, Table 3). On single-target searches, saccades to dissimilar distractors were faster than those to similar distractors and targets. On dual-target searches, mean saccadic latencies to dissimilar distractors and targets were almost identical. A repeated-measures ANOVA yielded a significant main effect of object type, *F*(2,22) = 3.79, *p* = 0.038, *η*_p_^2^ = .26, and a significant Search by Object type interaction, *F*(2,22) = 8.44, *p* = 0.002, *η*_p_^2^ = .43. The main effect of search did not reach statistical significance, *F*(1,11) = 4.57, *p* > 0.05, *η*_p_^2^ = .29, *Post hoc* tests revealed median saccadic latencies to dissimilar distractors were significantly faster on single- than dual-target searches (*M* = 103ms, *p* < 0.001), while differences to targets (*M* = 24ms, *p* > 0.05) and similar distractors (*M* = 1ms, *p* > 0.05) were not statistically significant.

#### Model estimates and fits

**Fig. 6:**
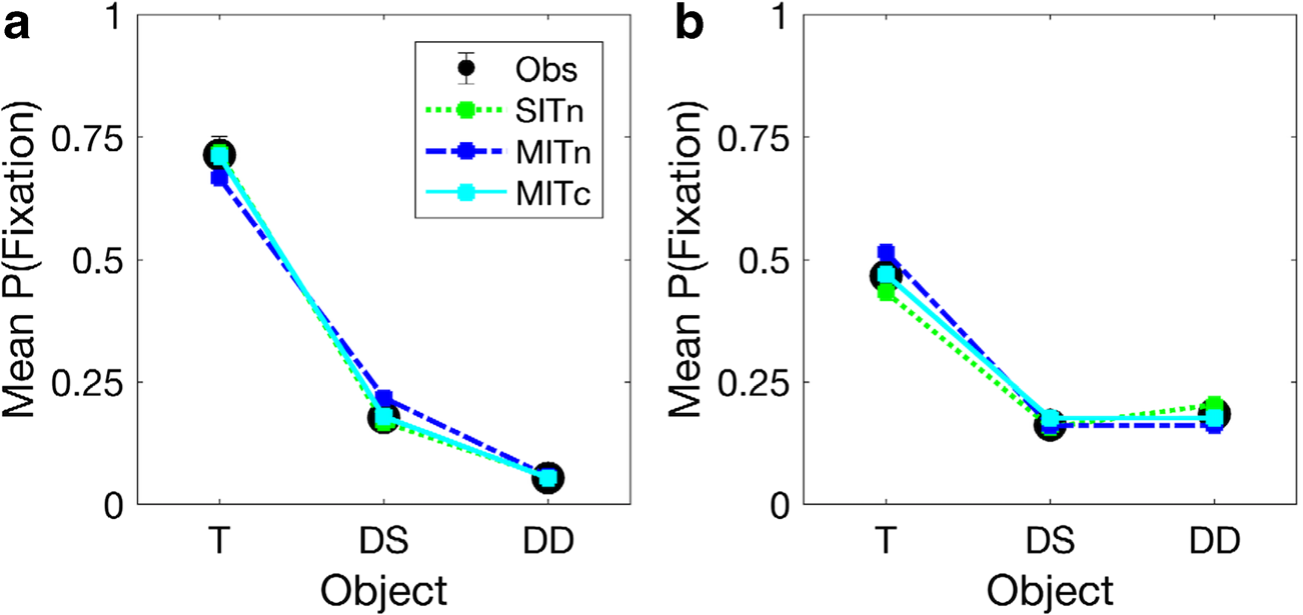
Mean proportion of observed (Obs) and estimated fixations to targets (T), similar (DS) and dissimilar (DD) distractors on single-target (**a**) and dual-target (**b**) searches in Experiment 2. *SITn* single-item template, noise-limited, *MITn* multiple-item template, noise-limited, *MITc* multiple-item template, capacity-limited model. Error bars denote standard errors of observed means

Figure 6 plots the mean observed and predicted proportions of fixations to targets and distractors on single- and dual-target searches. As can be seen, all three models produce reasonable fits to the data, with SIT and MIT models capturing the difference in the discriminability of targets against similar and dissimilar distractors on single-target searches, as well as the increase in the likelihood of fixating dissimilar distractors on dual-target searches. All three models also capture the marked decrease in the accuracy of first saccades on dual- compared to single-target searches. For single-target searches, the MIT, noise-limited model underestimates the proportion of fixations to targets and overestimates those to similar distractors. The same model also overestimates the likelihood of fixating the target on dual-target searches. Fits for the SIT, noise-limited and MIT, capacity-limited models are much closer to the observed data. For the former, mean estimates fall within 1 standard error of the observed means for all but dissimilar distractors on dual-target searches. For the latter, mean estimates fall within 1 standard error of observed means for all objects across both types of search. These differences are reflected in the statistical comparisions listed in Table 4, which yield smaller summed AIC values and higher fit indices for the MIT, capacity-limited than the SIT and MIT, noise-limited models. Fit indices also favour the MIT, capacity-limited model at the individual level, where AIC values were smaller and fit indices larger compared to the SIT and MIT, noise-limited models for 8/12 observers. The mean absolute difference between observed and estimated data was also smaller for the MIT-capacity (0.02) than the MIT, noise-limited (0.03) and SIT, noise-limited models (0.03; individual parameter estimates and goodness-of-fit statistics are presented in Tables S2a, S2b and S2c in the ESM).

**Table 4:** Mean parameter estimates for the SIT, noise-limited (SITn), MIT, noise-limited (MITn) and MIT, capacity-limited- (MITc) models in Experiment 2. Standard errors of means are reported in parentheses and *d’* subscripts S and D denote similar and dissimilar distractors, respectively

Model	*d’*_*S*_	*d*’_*D*_	Fit	∑ AIC
SITn	-1.28 (.18)	-1.93 (.15)	.22 (.09)	382.96
MITn	-0.96 (.13)	-1.87 (.16)	.09 (.03)	392.78
MITc	-1.20 (.16)	-1.98 (.17)	.44 (.09)	331.22

*SITn* single-item template, noise-limited, *MITn* multiple-item template, noise-limited, *MITc* multiple-item template, capacity-limited model

### Discussion

The results of Experiment 2 reveal a significant reduction in the accuracy of first saccades on dual- compared to single-target searches. This finding is consistent with previous evidence for a decrease in saccadic selectivity during dual-target searches for colour-defined targets (Stroud, Menneer, Cave, Donnelly, & Rayner, 2011; but see Beck et al., 2012). In the current study, changes in the proportions of fixations to objects classified in terms of their similarity to the target(s) provide an index of the localised selection that guides search. On single-target searches, 89% of fixations were made to objects that grouped with the cue; 71% to the target and 18% to the similar distractor. Saccades to each of the remaining dissimilar distractors averaged just 5%, indicating observers’ ability to select objects with similar over dissimilar orientations to the cue during search. On dual-target searches, 63% of saccades were made to objects that grouped with the cued-target that was presented in the display, 47% to the target and 16% to the similar distractor. Saccades to each of the dissimilar distractors increased to 19% of trials, a figure equivalent to that for similar distractors on single-target searches. In the current study, this equivalence is predicted by cue-based selection that reverses the similarity values assigned to leftward and rightward distractors during dual-target searches. Figure 1 illustrates this situation when Target-Left is cued and present in the display. Hypothetical similarity values for objects are plotted on the ordinate of panels B to D. Observers saccade to the location with the maximum value, with competition for selection reflecting the similarity between the cue and objects in the display. On a single target search (Panel B), competition is largest between the cued target (TL) and its similar distractor (DL). On a dual-target search (Panel D), activation associated with the template for the rightward leaning target (TR) prioritises the complementary subset of distractors, which generates equivalent competition from similar (DL) and dissimilar distractors (DR) during saccadic targeting.

Figure 6 shows all three models provide reasonable fits to the observed distribution of saccades on single- and dual-target searches at the group level. Comparisons indicate better fit indices for the MIT, capacity-limited than the MIT, noise-limited model, where *d*’ is independent of the number of comparisons required to localise the target. Group and individual comparisons also reveal better fit indices for the MIT, capacity-limited than the SIT, noise-limited model, supporting saccadic targeting based on separate similarity distributions for each cue. As with Experiment 1, comparisons of the fit indices for SIT and MIT models support a dual-target cost that is (1) best approximated by an inverse relationship between accuracy and the product of set size and the number of cued orientations, and (2) exceeds that predicted by an increase in decision-noise alone during search for independently cued targets. The analysis of saccadic latencies also rules out a general slowing or speed-accuracy trade off on dual- compared to single-target searches (Godwin, Walenchcok, Houpt, Hout & Goldinger, 2015). Surprisingly, mean saccadic latencies to targets and distractors were slower on dual- than single-target searches, raising the possibility that observers require less evidence to initiate saccades when similarity distributions contain maxima for independently cued targets. Notably, however, the reduction for targets and similar distractors was not statistically significant, indicating any change in decision thresholds for these objects is relatively small. Despite the lack of reliable changes in latencies for these objects, our analyses did reveal a statistically significant increase in saccadic latencies to dissimilar distractors on dual- compared to single-target searches. This is likely to reflect first fixations to dissimilar distractors that were initiated before top-down selection was fully implemented during search, as well as an increase in the competitive weights associated with dissimilar distractors during dual-target search (van Zoest, Donk, & Theeuwes, 2004). Taken together, the accuracy and latency data are consistent with cue-related changes in the likelihood and latency of fixations to leftward and rightward leaning distractors when searching for two targets selects complementary sets of similarity distributions for objects in the display.

## General discussion

The current study was designed to investigate the nature of the dual-target cost on perceptual decisions and saccadic targeting during covert and overt search. To do this, we applied SIT and MIT SDT models to predict observers’ accuracy when comparisons between visual input and VSTM are limited to (1) a single-item attentional template or (2) multiple-item templates for independently cued targets during dual-target search. In addition, we compared observers’ accuracy to noise- and capacity-limited derivations for both models of attentional control. Each assumes search entails the comparison of noisy internal representations of visual input with one or two cued-orientations. Noise-limited derivations model the dual-target cost as an increase in stochastic noise when the number of comparisons doubles on dual- compared to single-target searches. Capacity-limited derivations apply an inverse square-root relationship to model an additional dependency between discriminability and the number of comparisons during search (Corbett & Smith, 2017; Eckstein et al., 2000; Shaw, 1980; Palmer, 1994). Each provides a means to generalise EEG evidence for parallel top-down activation of independently cued features (Christie, Livingstone, & McDonald, 2015; Grubert & Eimer, 2015) to behavioural data by incorporating (1) decision-noise and (2) the distribution of resources during the evaluation of evidence for one of two potential targets during dual-target search.

Experiment 1 contrasted the accuracy of perceptual decisions in brief displays during single- and dual-target searches. Display durations of less than 100 ms were used to impose a common temporal constraint and preclude eye movements on both types of search. Comparisons between the observed and estimated probability distributions of hits and false alarms revealed better fits for the MIT than SIT models, and a better fit for the MIT, capacity- than MIT, noise-limited model. The close approximation between observed and estimated data for the former indicates the dual-target cost is consistent with an increase in decision-noise and the distribution of capacity-limited decision processes when observers compare visual input with independently cued target values. This interpretation supports previous evidence for the parallel selection of independently cued features during dual-target search (Eimer & Grubert, 2014; Grubert & Eimer, 2015), and suggests the dual-target cost reflects a quantitative rather than a qualitative reduction in the accuracy of perceptual decisions (Barrett & Zobay, 2014; Hayward, Walenchok, Houpt, Hout & Goldinger, 2015; Irons et al., 2012). Modelling the dual-target cost as an inverse function of the number of comparisons required to classify the display provides a mechanistic account of the discrepancy between electrophysiological and behavioural markers of the dual-target cost (Christie et al., 2015; Grubert & Eimer, 2015) that is consistent with theoretical accounts of resource distribution during guided search (Barrett, Shimozaki, Jensen, & Zobay, 2016; Corbett et al., 2017; Eckstein et al., 2000; Eckstein, 2011; Palmer, 1994; Põder, 2017).

Experiment 2 adapted the SDT models for Experiment 1 to a 4-AFC task in which the accuracy of first fixations was the dependent measure. The data revealed a significant decrease in the accuracy of initial fixations during dual- compared to single-target searches, extending findings from free-view tasks (e.g. Stroud et al., 2012). In our data, the dual-target cost reflects a marked change in the distribution of fixation probabilities to targets and distractors during single- and dual-target search (see Fig. 5). On single-target searches, observers made very few (~5%) saccades to dissimilar distractors. These erroneous fixations had shorter latencies than those to targets or similar distractors, illustrating the near exclusion of dissimilar distractors as inspection time increased. On dual-target searches, the requirement to evaluate evidence for both targets elicited a more equal distribution of first fixations to leftward and rightward distractors. Assuming first fixations provide an index of latent selection during search, changes in the distribution of fixations evidence cue-related changes in the weights assigned to subsets of distractors during single- and dual-target searches. Comparisons between the observed and predicted distribution of fixations indicated SIT and MIT models provide a good approximation of observers’ performance on single- and dual-target searches. Comparisons between models indicated the MIT models produced the closest fit between observed and estimated data for 8/12 observers. Scaling *d*’ by a factor of the square root of the product of cued orientations and set size produced better fits than fixing *d*’ to a single value in 7/8 observers whose data were best approximated by MIT models. These results are consistent with a dual-target cost for most observers that is commensurate with an increase in decision-noise and the distribution of capacity-limited resources when saccadic targeting is informed by multiple-item attentional templates (Sewell et al., 2014). A comparison of saccadic latencies also revealed a significant decrease on dual- compared to single-target searches, which was specific to distractors dissimilar to the target presented in the display. These data support cue-related changes in the selection-weights assigned to subsets of distractors on dual-target searches, and illustrate comparable saccadic latencies to objects based on their similarity to independently cued orientations.

The current results are difficult to reconcile with a fixed single-item limit on top-down selection during search. For most observers, our data support perceptual decisions and saccadic targeting that are informed by independently cued features during dual-target search. For a minority of observers, however, perceptual decisions and saccadic targeting were best approximated by SIT models that restrict the comparison of visual input to a single cued item during search (Olivers et al., 2011; Houtkamp & Roelfsema, 2006, 2009). This inter-subject variability suggests behavioural indices of the dual-target cost may reflect distinct post-selection strategies during the evaluation of evidence for one of two cued targets. In our models, *d’*s representing the similarity between cued orientations and objects in the display are fixed. Changes in accuracy predicted by each model therefore reflect changes in the way selected information is evaluated during single- and dual-target searches, rather than changes to localised responses that signal target features in the display (Cohen et al., 2009; Eimer, 1996; Luck & Hillyard, 1994). In terms of the theoretical distinction between guidance and target-identification during search (e.g. Castelhano, Pollatsek, & Cave, 2008; Hout & Goldinger, 2015; Palmer, 1995), our analyses indicate the dual-target cost can be explained by a decrease in the accuracy of target-identification as well as changes in way selected information is compared to the attentional template during search. Determining the internal (i.e., VSTM capacity) and external (i.e., number of cued values, object complexity and target-distractor discriminability) factors that influence these strategies remains an important question for future research. A relevant distinction between this and previous studies of the dual-target cost is our use of targets that were matched in terms of perceptual similarity to numerically equivalent subsets of distractors. This design negates potential stimulus-driven asymmetries in the similarity distributions elicited by separate cues, which may have biased selection and evaluation in previous studies toward the most discriminable (or memorable) categorical target (i.e., Houtkamp & Roelfsema, 2009; Mestry, Menneer, Cave, Godwin, & Donnelly, 2017).

Evidence that observers can evaluate evidence for independently cued targets in our study provides insights into the way decision-noise and capacity limits affect search accuracy in brief displays or during initial saccadic targeting. Our findings, however, are insensitive to dual-target costs that may arise during subsequent saccadic sampling in free-view displays. Previous eye-movement data suggest observers can switch between simultaneous and successive modes of guidance during dual-target searches (Beck et al., 2012; Cave et al., 2018). In this situation, the reduction in the accuracy of saccades appears to reflect an increased number of fixations to objects dissimilar to both cues (Stroud et al., 2012). Whether this cost is commensurate with that evidenced in the current study, or reflects additional constraints associated with the withdrawal of resources from one attentional template during the planning of successive saccades (Rajsic et al., 2017), or the requirement to retain the locations and status of previously fixated objects against multiple decision criteria, remain important questions for future research.

Our results suggest an important determinant of behavioural measures of the dual-target cost is the requirement to distribute post-selection resources during the comparison of visual input with independently cued features in VSTM (Sewell et al., 2014; Smith & Sewell, 2013). In the current study, displays contained a maximum of four objects, a number that corresponds to estimates of VSTM capacity (see Fukuda, Awh, & Vogel, 2010). Notably, however, evaluating four objects against two target templates required eight comparisons, which exceeds VSTM estimates on most tasks. Recent evidence from hybrid search has shown observers can successfully detect one of 100 potential targets encoded in long-term memory (Drew, Boettcher, & Wolfe, 2017). Eye movements during hybrid search reveal a logarithmic relationship between dwell time and the number of potential targets, which is consistent with the comparison of visual input with multiple memory representations within a single fixation. Findings from hybrid search highlight potential limits on the timing and accuracy of successive fixations during search for categorical objects in long-term memory. Our results quantify the dual-target cost on the evaluation of independently cued feature values during perceptual decisions in the absence of eye movements and on the accuracy of first saccades during overt search. The ability of the SIT and MIT models to predict observers’ performance across these tasks with relatively few free parameters suggests they provide a theoretically plausible description of attentional selection and the decision processes underlying the initial stages of dual-target search. As such, our results provide a bridge between electrophysiological markers of top-down activation for independently cued features and behavioural measures of the dual-target cost, and provide important insights into the relationship between objects in VSTM, attentional control and decision processes during covert search and initial saccadic targeting in free-view displays.

## Electronic supplementary material

ESM 1 (DOCX 183 kb)

## Data Availability

Experiments reported in the manuscript were not preregistered. Summary data for Experiments 1 and 2 are available at: 10.25392/leicester.data.7955969.
