# Supplementary material for: Concurrent evaluation of independently cued features during perceptual decisions and saccadic targeting in visual search
Source: Atten Percept Psychophys. 2019 Sep 9;82(3):966–84. doi: 10.3758/s13414-019-01854-w (PMC7303085; doi:10.3758/s13414-019-01854-w)
Supplement: Supplementary file 1 — (DOCX 183 kb) [file 13414_2019_1854_MOESM1_ESM.docx]

Supplementary Information

***Table S1a****. Individual parameter estimates and fit-statistics for the 1-Template, Noise-Limited SDT model in Experiment 1. Row entries represent a single observer.*

| *λ* by No. Comparisons | | |  | | | | |
| --- | --- | --- | --- | --- | --- | --- | --- |
| ST1 | ST2 | ST4 | *d*’_1_ | *d*’_2_ | Fit 1 | Fit 2 | AIC |
| -0.70 | -0.02 | 0.19 | -1.66 | -1.36 | 0.00 | 0.05 | 89.72 |
| -1.20 | -0.51 | 0.03 | -2.46 | -0.93 | 0.00 | 0.06 | 95.40 |
| -1.67 | -0.87 | -0.51 | -2.06 | -1.44 | 0.01 | 0.05 | 81.19 |
| -1.40 | -0.63 | -0.20 | -2.61 | -1.50 | 0.00 | 0.05 | 88.47 |
| -1.76 | -0.94 | -0.43 | -3.34 | -1.49 | 0.00 | 0.10 | 142.28 |
| -1.92 | -0.95 | -0.62 | -3.17 | -1.54 | 0.00 | 0.08 | 111.05 |
| -1.85 | -0.92 | -0.72 | -2.98 | -1.27 | 0.00 | 0.10 | 200.32 |
| -1.20 | -0.37 | -0.02 | -1.76 | -0.95 | 0.00 | 0.07 | 89.40 |
| -1.60 | -0.70 | -0.48 | -1.67 | -1.15 | 0.00 | 0.05 | 82.33 |
| -1.42 | -0.72 | -0.43 | -2.29 | -1.79 | 0.00 | 0.06 | 94.42 |
| -1.01 | -0.37 | -0.11 | -1.73 | -0.73 | 0.00 | 0.07 | 97.79 |

***Table S1b****. Individual parameter estimates and fit-statistics for the 1-Template, Capacity-Limited SDT model in Experiment 1. Row entries represent a single observer.*

| *λ* by No. Comparisons | | |  | | | | |
| --- | --- | --- | --- | --- | --- | --- | --- |
| ST1 | ST2 | ST4 | *d*’_1_ | *d*’_2_ | Fit 1 | Fit 2 | AIC |
| -0.98 | -0.62 | -0.52 | -1.97 | -2.23 | 0.01 | 0.05 | 82.27 |
| -1.77 | -1.44 | -1.05 | -3.01 | -1.95 | 0.14 | 0.04 | 74.45 |
| -2.13 | -1.54 | -1.26 | -2.50 | -2.20 | 0.00 | 0.06 | 91.41 |
| -1.91 | -1.56 | -1.32 | -3.05 | -2.59 | 0.00 | 0.05 | 84.67 |
| -2.51 | -2.22 | -1.94 | -3.99 | -2.88 | 0.00 | 0.09 | 135.79 |
| -2.92 | -2.30 | -2.17 | -4.10 | -3.00 | 0.00 | 0.07 | 104.78 |
| -3.06 | -2.37 | -2.33 | -4.17 | -2.81 | 0.00 | 0.08 | 167.61 |
| -1.62 | -1.02 | -0.75 | -2.17 | -1.68 | 0.02 | 0.05 | 79.63 |
| -2.17 | -1.40 | -1.25 | -2.26 | -1.93 | 0.24 | 0.04 | 72.75 |
| -2.22 | -1.74 | -1.62 | -3.11 | -3.02 | 0.12 | 0.04 | 73.25 |
| -1.31 | -0.95 | -0.76 | -2.03 | -1.38 | 0.00 | 0.06 | 86.37 |

***Table S1c****. Individual parameter estimates and fit-statistics for the 2-Template, Noise-Limited SDT model in Experiment 1. Row entries represent a single observer.*

| *λ* by No. Comparisons | | | |  |  |  |  |  |
| --- | --- | --- | --- | --- | --- | --- | --- | --- |
| ST1 | ST2/DT1 | ST4/DT2 | DT4 | *d*’_1_ | *d*’_2_ | Fit 1 | Fit 2 | AIC |
| -0.79 | 0.10 | 0.39 | 0.86 | -1.67 | -1.05 | 0.00 | 0.05 | 87.55 |
| -1.79 | -0.24 | 0.20 | 0.46 | -2.95 | -0.75 | 0.05 | 0.04 | 78.81 |
| -1.65 | -0.62 | -0.18 | 0.20 | -1.95 | -1.08 | 0.00 | 0.05 | 86.71 |
| -1.58 | -0.44 | -0.04 | 0.31 | -2.64 | -1.34 | 0.12 | 0.04 | 75.94 |
| -1.84 | -0.81 | -0.44 | -0.18 | -3.23 | -1.62 | 0.00 | 0.07 | 102.87 |
| -2.31 | -0.70 | -0.42 | -0.05 | -3.36 | -1.41 | 0.00 | 0.06 | 93.93 |
| -4.12 | -0.79 | -0.29 | -0.53 | -5.00 | -1.25 | 0.00 | 0.04 | 94.54 |
| -1.66 | -0.17 | 0.25 | 0.44 | -2.15 | -0.66 | 0.78 | 0.02 | 70.13 |
| -2.05 | -0.47 | 0.08 | 0.26 | -2.04 | -0.59 | 0.03 | 0.05 | 78.76 |
| -2.27 | -0.32 | -0.02 | 0.28 | -3.07 | -1.24 | 0.03 | 0.04 | 76.75 |
| -1.25 | -0.17 | 0.09 | 0.33 | -1.84 | -0.62 | 0.00 | 0.06 | 86.86 |

***Table S1d****. Individual parameter estimates and fit-statistics for the 2-Template, Capacity-Limited SDT model in Experiment 1. Row entries represent a single observer.*

| *λ* by No. Comparisons | | | |  |  |  |  |  |
| --- | --- | --- | --- | --- | --- | --- | --- | --- |
| ST1 | ST2/DT1 | ST4/DT2 | DT4 | *d*’_1_ | *d*’_2_ | Fit 1 | Fit 2 | AIC |
| -0.86 | -0.68 | -0.55 | -0.10 | -1.81 | -4.81 | 0.01 | 0.04 | 78.40 |
| -1.78 | -1.36 | -1.16 | -0.96 | -2.93 | -2.17 | 0.35 | 0.03 | 72.68 |
| -1.74 | -1.66 | -1.33 | -0.96 | -2.06 | -5.06 | 0.14 | 0.04 | 76.71 |
| -1.56 | -1.54 | -1.39 | -1.12 | -2.60 | -5.60 | 1.00 | 0.01 | 65.04 |
| -1.87 | -2.21 | -2.14 | -2.00 | -3.27 | -6.27 | 0.09 | 0.04 | 74.43 |
| -2.52 | -2.15 | -2.19 | -1.95 | -3.60 | -3.43 | 0.64 | 0.02 | 66.66 |
| -3.07 | -2.33 | -2.14 | -2.39 | -4.02 | -3.09 | 0.19 | 0.03 | 76.09 |
| -1.61 | -1.07 | -0.81 | -0.67 | -2.08 | -2.03 | 0.84 | 0.02 | 70.23 |
| -2.02 | -1.46 | -1.07 | -0.93 | -2.00 | -2.23 | 0.24 | 0.03 | 73.53 |
| -2.15 | -1.47 | -1.46 | -1.27 | -2.93 | -3.01 | 0.37 | 0.03 | 70.60 |
| -1.26 | -1.10 | -1.00 | -0.81 | -1.85 | -2.34 | 0.27 | 0.04 | 75.92 |

***Table S2a****. Individual parameter estimates and fit-statistics for the 1-Template, Noise-Limited SDT model in Experiment 2. Row entries represent a single observer.*

| *d*’_1_ | *d*’_2_ | Fit 1 | Fit 2 | AIC |
| --- | --- | --- | --- | --- |
| -1.88 | -2.03 | 0.15 | 0.02 | 28.37 |
| -1.89 | -2.32 | 0.00 | 0.04 | 35.06 |
| -0.52 | -1.74 | 0.24 | 0.02 | 28.29 |
| -0.57 | -1.85 | 0.80 | 0.01 | 26.17 |
| -2.16 | -2.55 | 0.00 | 0.07 | 58.59 |
| -0.49 | -1.16 | 0.18 | 0.02 | 29.38 |
| -1.54 | -2.41 | 0.04 | 0.03 | 31.60 |
| -0.55 | -1.25 | 0.13 | 0.02 | 29.94 |
| -0.96 | -1.22 | 0.92 | 0.01 | 26.14 |
| -1.30 | -2.33 | 0.05 | 0.03 | 30.53 |
| -2.01 | -2.52 | 0.10 | 0.02 | 28.59 |
| -1.50 | -1.80 | 0.06 | 0.03 | 30.31 |

***Table S2b****. Individual parameter estimates and fit-statistics for the 2-Template, Noise-Limited SDT model in Experiment 2. Row entries represent a single observer.*

| *d*’_1_ | *d*’_2_ | Fit 1 | Fit 2 | AIC |
| --- | --- | --- | --- | --- |
| -1.44 | -1.77 | 0.01 | 0.05 | 39.10 |
| -1.32 | -2.34 | 0.01 | 0.04 | 33.17 |
| -0.42 | -1.72 | 0.14 | 0.03 | 29.11 |
| -0.42 | -1.76 | 0.03 | 0.04 | 32.63 |
| -1.63 | -2.58 | 0.28 | 0.02 | 25.96 |
| -0.37 | -1.09 | 0.02 | 0.04 | 34.26 |
| -1.12 | -2.42 | 0.02 | 0.04 | 33.38 |
| -0.46 | -1.22 | 0.16 | 0.02 | 29.84 |
| -0.80 | -1.14 | 0.35 | 0.02 | 28.11 |
| -0.97 | -2.33 | 0.03 | 0.04 | 32.76 |
| -1.34 | -2.44 | 0.00 | 0.05 | 42.21 |
| -1.21 | -1.63 | 0.01 | 0.03 | 32.27 |

***Table S2c****. Individual parameter estimates and fit-statistics for the 2-Template, Capacity-Limited SDT model in Experiment 2. Row entries represent a single observer.*

| *d*’_1_ | *d*’_2_ | Fit 1 | Fit 2 | AIC |
| --- | --- | --- | --- | --- |
| -1.82 | -1.95 | 0.18 | 0.01 | 27.88 |
| -1.67 | -2.49 | 0.92 | 0.00 | 24.14 |
| -0.53 | -1.76 | 0.71 | 0.01 | 26.37 |
| -0.52 | -1.81 | 0.14 | 0.02 | 30.03 |
| -2.01 | -2.80 | 0.02 | 0.03 | 32.26 |
| -0.49 | -1.15 | 0.13 | 0.03 | 30.06 |
| -1.40 | -2.55 | 0.65 | 0.01 | 25.04 |
| -0.56 | -1.28 | 0.49 | 0.01 | 27.78 |
| -0.96 | -1.22 | 0.87 | 0.01 | 26.29 |
| -1.21 | -2.43 | 0.69 | 0.01 | 25.33 |
| -1.73 | -2.59 | 0.36 | 0.02 | 25.81 |
| -1.48 | -1.77 | 0.09 | 0.02 | 30.24 |
